# The clinical heterogeneity of drug-induced myoclonus: an illustrated review

**DOI:** 10.1007/s00415-016-8357-z

**Published:** 2016-12-16

**Authors:** Sabine Janssen, Bastiaan R. Bloem, Bart P. van de Warrenburg

**Affiliations:** 1 Department of Neurology 935, Radboud University Medical Center, Donders Institute of Brain, Cognition and Behaviour, P.O. Box 9101, 6500 HB Nijmegen, The Netherlands; 2 Biomedical Signal and Systems Group, MIRA Institute for Biomedical Technology and Technical Medicine, University of Twente, P.O. 217, 7500 AE Enschede, The Netherlands

**Keywords:** Drug-induced myoclonus, Myoclonus/phenotype, Myoclonus/physiopathology

## Abstract

A wide variety of drugs can cause myoclonus. To illustrate this, we first discuss two personally observed cases, one presenting with generalized, but facial-predominant, myoclonus that was induced by amantadine; and the other presenting with propriospinal myoclonus triggered by an antibiotic. We then review the literature on drugs that may cause myoclonus, extracting the corresponding clinical phenotype and suggested underlying pathophysiology. The most frequently reported classes of drugs causing myoclonus include opiates, antidepressants, antipsychotics, and antibiotics. The distribution of myoclonus ranges from focal to generalized, even amongst patients using the same drug, which suggests various neuro-anatomical generators. Possible underlying pathophysiological alterations involve serotonin, dopamine, GABA, and glutamate-related processes at various levels of the neuraxis. The high number of cases of drug-induced myoclonus, together with their reported heterogeneous clinical characteristics, underscores the importance of considering drugs as a possible cause of myoclonus, regardless of its clinical characteristics.

**Electronic supplementary material:**

The online version of this article (doi:10.1007/s00415-016-8357-z) contains supplementary material, which is available to authorized users.

## Introduction

Myoclonus are involuntary sudden, brief, shock-like ‘jerky’ movements due to muscular contractions (‘positive myoclonus’) or sudden lapses of muscle contraction in active muscles (‘negative myoclonus’ or ‘asterixis’) [40, 44]. Myoclonus can be classified by distribution (focal, segmental, multifocal, and generalized) [75], by localization of the ‘pulse generator’ (cortical, subcortical, brainstem, spinal, or peripheral) [44], and by aetiology (physiological, essential, epileptic, symptomatic, and psychogenic) [44, 52]. In this paper, we review the phenomenon of drug-induced myoclonus, a subgroup of symptomatic myoclonus, with an emphasis on the clinical and pathophysiological heterogeneity of this phenomenon, which could mislead clinicians and result in insufficient consideration of drugs as the cause of myoclonus.

We first describe two personally observed cases of drug-induced myoclonus. Next, we present the results of our literature search on those drugs reported to cause myoclonus, including details of the corresponding clinical phenotype and, whenever available, data on the neuro-anatomical origins and pathophysiological processes.

## Case description

### Case A

Patient A was a 79-year-old woman who developed her first ever epileptic seizure 2 days after the start of intravenous penicillin and ciprofloxacin prescribed for pneumonia. On neurological examination after the seizure she was alert but showed jerky movements of trunk, abdomen, and arms (particularly the right shoulder) more than of her legs that she could not suppress (video 1). The spread and temporal gradient of these jerks were particularly indicative of propriospinal myoclonus. EEG showed no epileptic phenomena, not even at the time when the movements occurred during recording. Antibiotic therapy was switched to claritromycin and ceftazidim, after which the myoclonus disappeared. The final diagnosis was that of ciprofloxacin and/or penicillin-induced propriospinal myoclonus.

### Case B

Patient B was a 66-year-old man who had been diagnosed 12 years previously with Parkinson’s disease for which he took levodopa/benserazide 125 mg t.i.d. plus 125 mg b.i.d. as dispersible tablets, and ropinirole 6 mg b.i.d. His medical history reported a left-sided stereotactic thalamotomy because of a troublesome tremor of his right hand and a cervical disc herniation. Because of peak-dose dyskinesias and marked off periods during the night, amantadine was started and augmented to 100 mg t.i.d. Slow-release levodopa/benserazide ante noctum was added to the treatment regimen. One month after these treatment adjustments, the patient developed involuntary jerks through his whole body but predominantly in his face and neck, also severely affecting his speech. These jerks were not sensitive to stimuli and occurred mainly during action (both positive and negative) but were also present at rest. During walking, axial action myoclonus was apparent (video 2). The remaining examination showed an asymmetric hypokinetic-rigid syndrome, the severity of which was similar to prior examination. Suspecting drug-induced generalized myoclonus, amantadine was tapered off, and the myoclonus disappeared within 2 weeks.

## Methods

### Search strategy

We searched PubMed using the MeSH terms “Myoclonus/chemically induced”, “Myoclonus/etiology”, “Myoclonus/pharmacology”, “Myoclonus/physiology”, “Myoclonus/physiopathology”, “Drug-Related Side Effects and Adverse Reactions”, and “Dyskinesia, Drug-Induced”. Only articles in English, published before September 2016, were reviewed for relevance.

## Results

Our literature search on drug-induced myoclonus mainly identified case reports and (mostly small) case series (the largest involving 32 patients). Table 1 summarizes the number of cases reported per subclass of drugs associated with myoclonus, the distribution of myoclonus, and one or two relevant references per category. The full table with all references considered (Table 2) is available as supplementary material. Almost all classes of drugs have been linked to myoclonus. The clinical phenotype covered the whole spectrum, from a focal to a generalized distribution. The presumed anatomic structures and neurotransmitters involved are suggested to differ per causative agent. Drug-induced myoclonus was usually reversible following withdrawal of the offending drug [10, 44], and only a single case of persistent myoclonus has been reported [75]. We here describe the characteristics of myoclonus caused by the four classes of drugs most often reported in relation to myoclonus (opiates, antidepressants, antipsychotics, and antibiotics) and by the group of drugs involved in our case B (NMDA antagonists).

**Table 1:** Case reports and illustrative references upon medication-induced myoclonus

Pharmacological class	Pharmacological subclass	Number of cases reported ^Illustrative reference(s)^					
		All distributions	Focal	Segmental	Multifocal	Generalized	Distribution not described
Opiates	Full agonists	105	1 [47]	–	18 [32]	13 [19]	73 [7, 83 78 ]
	Partial agonist–antagonist	7	–	–	–	–	7 [7]
Antidepressants	Selective serotonin-reuptake inhibitors (SSRIs)	44	–	–	2 [30]	6 [86]	36 [74]
	Tricyclic antidepressants (TCAs)	55	5 [54]	–	2 [20]	4 [68]	44 [7, 8]
	Lithium	10	–	–	6 [11, 20]	2 [14]	2 [7]
	Monoamine oxidase (MAO) inhibitors	4	–	–	1 [5]	–	3 [7]
	Serotonin-norepinephrine reuptake inhibitor (SNRI)	1	–	–	–	1 [18]	–
	Noradrenalin and dopamine reuptake inhibitors	1	1 [31]	–	–	–	–
Antipsychotics	Typical	65	–	–	56 [93]	1 [16]	8 [7]
	Atypical	15	–	–	5 [6]	3 [92]	7 [7]
Antibiotics	β-lactams	40	–	–	3 [80]	3 [87]	34 [7, 79]
	Quinolones	34	–	–	2 [81]	2 [21]	30 [7]
	Sulfonamides	3	–	–	2 [41]	1 [58]	–
	Aminoglycosides	6	–	–	–	1 [75]	5 [7]
Anxiolytics	Benzodiazepines	66	–	–	7 [51]	–	59 [7]
Anti-epileptics	Gabapentin	27	3 [4]	–	17 [4, 101]	3 [77, 101]	4 [7]
	Pregabalin	9	1 [35]	–	8 [63]	–	–
	Valproic acid	10	–	–	–	1 [98]	9 [1, 7]
	Lamotrigine	7	–	–	3 [23]	1 [13]	3 [74]
	Carbamazepine	5	1 [50]	–	–	–	4 [7, 27]
	Phenytoine	4	–	–	–	2 [17]	2 [7]
	Topiramate	4	2 [45]	–	1 [3]	1 [64]	–
	Phenobarbital	2	–	–	–	–	2 [7]
	Vigabatrin	2	–	–	2 [62]	–	–
	Clobazam	1	–	–	–	–	1 [27]
Anti-parkinsonians	L-dopa	28	–	–	–	–	28 [7, 43, 90]
	Dopamine agonists	8	–	–	–	–	8 [7, 90]
	Non-competitive (NMDA)-glutamatereceptor-antagonist (amantadine) (also see ‘anti-dementia’)	10	2 [31]	–		1 [96]	7 [55]
	COMT inhibitors	1	–	–	–	–	1 [7]
	MAO-inhibitors	1	–	–	–	–	1 [7]
Anesthetics	General anesthetics	42	1 [89]	15 [97]	8 [97]	7 [97, 46]	11 [7]
	Local anesthetics	4	–	–	4 [2]	–	–
Anti-dementia	Cholinesterase inhibitors	18	–	–	–	–	18 [7]
	Non-competitive (NMDA)-glutamatereceptor-antagonist (memantine) (also see anti-parkinsonians)	9	–	1 [69]	–	3 [60]	5 [7, 66]
Cytostatics	Ifosfamide	5	–	–	1 [56]	4 [76]	–
	Prednimustine	4	–	–	3 [53, 59]	1 [53]	–
	Chlorambucil	2	–	–	1 [95]	–	1 [95]
Others	Anti-emetics	23	1 [36]	1 [61]	2 [12]	–	19 [7]
	Anti-arrhythmics	5	–	–	3 [91]	1 [84]	1 [7]
	Vitamins	5	–	–	4 [99]	1 [65]	–
	Anti-hypertensives	2	–	–	2 [88]	–	–
	Contrast agents	3	1 [24]	–	2 [9]	–	–
	Immunomodulating drugs	2	–	–	1 [22]	–	1 [7]
	Anti-fibrinolytic agents	1	–	–	1 [34]	–	–
	Anti-histamines	1	–	–	–	1 [37]	–
	Anti-hypotensives	1	–	–	–	–	1 [94]
	Anti-tussives	1	–	–	–	1 [82]	–
	Adrenergic bronchodilators	3	–	–	3 [57]	–	–
	NSAID	1	–	–	–	–	1 [7]
	Anti-viral agents	1	–	–	1 [28]	–	–
	Anti-malaria prophylaxis	1	–	–	1 [39]	–	–

Classes and subclasses of drugs described to cause drug-induced myoclonus. References were sorted to distribution of myoclonus. The numbers of reported cases of drug-induced myoclonus are listed. One or two illustrative reference(s) per distribution is/are listed in superscript

– No studies describing myoclonus with this distribution, for this class of drugs. The references used to count the number of cases reported are listed in Table 2 available as ‘supplementary material’

### Opiates

Myoclonus may occur as a result of initial administration, change, or withdrawal of opiates [19, 32, 47]. Mainly generalized, but also multifocal and a single case of focal myoclonus have been described (Table 1, and supplementary Table 2). Opiate-related myoclonus occurs more frequently in patients concurrently treated with antidepressant, antipsychotic, antiemetic, or nonsteroidal anti-inflammatory drugs [47]. The precise pathophysiology remains poorly understood. A neuro-excitatory effect of opioid compounds and metabolites has been attributed to glutamate activation of *N*-methyl-D-aspartate (NMDA) receptors, glycine-mediated disinhibition of neural pathways at the cortical or spinal level, antagonism of gamma-amino-butyric acid (GABA) activity in the spinal cord, serotonergic and GABAergic pathways in the brainstem, and dopaminergic pathways in the basal ganglia [32].

### Antidepressants

Various classes of antidepressants have been associated with myoclonus (Table 1, and supplementary Table 2). Selective serotonin-reuptake inhibitors (SSRIs) can cause multifocal [30, 67] or generalized myoclonus [48, 64, 86]. Tricyclic antidepressants (TCAs) can cause either focal (especially jaw) [26, 54], multifocal [20, 42], and generalized [14, 49, 68, 98] myoclonus. Lithium has been observed to cause multifocal [11, 20] and generalized [14] myoclonus. An EEG transient over the contralateral sensorimotor region preceding the myoclonus suggested a cortical origin of myoclonus in patients treated with a TCA [20] or lithium [11]. Serotoninergic mechanisms are probably involved in the generation of antidepressant-induced myoclonus [30]. While SSRIs increase serotonin levels in the synaptic cleft, TCAs increase serotonin activity, and lithium facilitates the presynaptic release of serotonin [20]. A combination of two serotonergic active drugs, such as a TCA and lithium, appears more likely to cause myoclonus than a single drug [14, 20].

### Antipsychotics

Classic antipsychotics, including haloperidol, have been reported to cause multifocal myoclonus of both arms, sensitive to posture [25, 85]; of limbs and of the face [16]; and of the trunk and limbs [93]. Atypical antipsychotics, including quetiapine and olanzapine, can cause both multifocal [33, 72] and generalized [29, 73, 92] myoclonus. The exact pathogenesis of antipsychotic-induced myoclonus has not yet been unraveled, but involvement of serotonergic [16, 72], dopaminergic [93], and GABA-ergic [92] mechanisms have all been suggested.

### Antibiotics

Antibiotic-induced myoclonus mainly occurs in association with high or toxic doses of antibiotics and/or underlying renal disease [75]. It is commonly accompanied by other symptoms, such as altered mental state, seizures (similar to our case A), aphasia, chorea, and skin rash [75]. Myoclonus due to β-lactam antibiotics clinically varies from subtle peri-ocular twitching to generalized myoclonus [75]. Myoclonus due to quinolones can be generalized [21, 38] or multifocal [81]. It is hypothesized that β-lactam antibiotics selectively antagonize [75] and quinolones completely inhibit [71] gamma aminobutyric acid (GABA) receptors, decreasing their inhibitory activity at nerve terminals, thus inducing a hyperexcitable neuronal state of the central nervous system that triggers myoclonus. Sulfonamides have been associated with multifocal and generalized myoclonus. A causative role of altered dopamine metabolism due to inhibition of dihydrofolate reductase [15] as well as increased phenylalanine levels due to the inhibition of phenylalanine metabolism have been proposed [41]. Multifocal myoclonus that is due to aminoglycosides has been attributed to NMDA receptor activation and subsequent excitotoxicity.

### NMDA antagonists

Myoclonus due to *N*-methyl-D-aspartate (NMDA) receptor antagonists has rarely been reported. Amantadine has been shown to reduce levodopa-induced dyskinesias [100] but paradoxically has also been reported to induce jaw myoclonus in two patients [31, 70] and generalized myoclonus in four patients [55, 96]. In addition, memantine gave rise to myoclonus in patients with dementia [58, 60, 66]. The mechanism underlying amantadine and memantine induced myoclonus remains unclear, but might involve altered levels of dopamine, serotonin, and/or glutamate release [55, 96].

## Conclusion

A French pharmacovigilance database study [7], registering all compulsorily reported adverse drug reactions in France, reported an incidence of drug-induced myoclonus of 0.2% (423/185.634 reported adverse events over a 20-year period), which might be an underestimation due to underreporting [7]. Our literature survey is not suitable to extract epidemiological data, but the large number of case reports that we identified does suggest that drug-induced myoclonus is not an uncommon phenomenon in movement disorder consultations. Of course, we cannot offer certainty about causality for the observed associations between drugs and myoclonus, which is inherent to a literature review of case reports and case series. However, in many cases, myoclonus appeared shortly after the prescription of a new (and presumably causally involved) drug, and disappeared again readily after this same drug was stopped, suggesting a causal relationship.

Our survey—as well as the French pharmacovigilance database study—found that the most important groups of drugs with links to myoclonus are: opiates, antidepressants, antipsychotic drugs, and antibiotics. However, drug-induced myoclonus may also be caused by a wide variety of other drugs. Drug-induced myoclonus is usually reversible upon discontinuation of the offending drug, and this stresses the importance of making the correct diagnosis of drug-induced myoclonus. Importantly, the phenomenology of the myoclonus can vary within a group of drugs and even for one particular drug, suggesting that the neuro-anatomical generator varies. From a clinical perspective, this also means that drugs as a cause cannot be discarded based solely on clinical myoclonus characteristics. The precise cellular and neurochemical alterations that make a certain drug cause myoclonus remain largely unclear and therefore need further study.

## Electronic supplementary material

Below is the link to the electronic supplementary material.

Supplementary material 1 (DOCX 215 kb)

Supplementary material 2 (DOCX 11 kb)

Supplementary material 3 (MP4 18275 kb)

Supplementary material 4 (MP4 11868 kb)
