# Supplementary material for: The clinical heterogeneity of drug-induced myoclonus: an illustrated review
Source: J Neurol. 2016 Dec 16;264(8):1559–66. doi: 10.1007/s00415-016-8357-z (PMC5533847; doi:10.1007/s00415-016-8357-z)
Supplement: Supplementary file 1 — Supplementary material 1 (DOCX 215 kb) [file 415_2016_8357_MOESM1_ESM.docx]

The clinical heterogeneity of drug-induced myoclonus**: an illustrated review**

**Supplementary material**

Table 2 Case reports that described medication-induced myoclonus.

| **Pharmacological class** | **Pharmacological subclass** | **Number of cases reported ^All reference(s)^** | | | | | |
| --- | --- | --- | --- | --- | --- | --- | --- |
|  |  | **All distributions** | **Focal** | **Segmental** | **Multifocal** | **Generalized** | **Distribution not described** |
| Opiates | Full agonists | 105 | 1 ^[77]^ | - | 18  ^[11, 17, 51, 52, 55, 66, 105, 108, 114]^ | ^­^13  ^[1, 13, 14, 16, 26, 27, 35, 36, 108, 110]^ | 73 ^[122], [15, 62, 73, 116, 121, 128]^ |
|  | Partial agonist - antagonist | 7 | - | - | - | - | 7^[15]^ |
| Antidepressants | Selective serotonin-reuptake inhibitors (SSRIs) | 44 | - | - | 2 ^[47, 104]^ | 6 ^[132]^ | 36 ^[15, 90, 113]^ |
|  | Tricyclic antidepressants (TCAs) | 55 | 5 ^[43, 88]^ | - | 2 ^[37, 68]^ | 4 ^[79, 80, 106]^ | 44 ^[15, 18, 43]^ |
|  | Lithium | 10 | - | - | 6 ^[20]; [37]^ | 2 ^[29]^ | 2 ^[15]^ |
|  | Monoamine oxidase (MAO) inhibitors | 4 | - | - | 1 ^[6]^ | - | 3 ^[15]^ |
|  | Serotonin-norepinephrine reuptake inhibitor (SNRI) | 1 | - | - | - | 1 ^[33]^ | - |
|  | Noradrenalin and dopamine reuptake inhibitors | 1 | 1 ^[50]^ | - | - | - | - |
| Antipsychotics | Typical | 65 | - | - | 56 ^[42, 130, 139]^ | 1 ^[31]^ | 8 ^[15]^ |
|  | Atypical | 15 | - | - | 5 ^[9, 57, 109, 126]^ | 3 ^[46, 112, 138]^ | 7^[8, 15]^ |
| Antibiotics | β-lactams | 40 | - | - | 3 ^[66, 85, 115, 124]^ | 3 ^[22, 75, 133]^ | 34 ^[15, 54, 69, 111, 123]^ |
|  | Quinolones | 34 | - | - | 2 ^[125]^ | 2 ^[38, 63]^ | 30^[15, 86]^ |
|  | Sulfonamides | 3 | - | - | 2 ^[30, 65]^ | 1 ^[93]^ | - |
|  | Aminoglycosides | 6 | - | - | - | 1 ^[117]^ | 5^[15]^ |
| Anxiolytics | Benzodiazepines | 66 | - | - | 7 ^[84, 100]^ | - | 59^[15]^ |
| Anti-epileptics | Gabapentin | 27 | 3 ^[5]^ | - | 17 ^[5, 7, 34, 53, 67, 147]^ | 3 ^[56, 120, 147]^ | 4 ^[15, 119]^ |
|  | Pregabalin | 9 | 1 ^[59]^ | - | 8 ^[53, 98, 131]^ | - | - |
|  | Valproic acid | 10 | - | - | - | 1 ^[145]^ | 9 ^[2, 15]^ |
|  | Lamotrigine | 7 | - | - | 3 ^[40]^ | 1 ^[25]^ | 3 ^[113]^ |
|  | Carbamazepine | 5 | 1 ^[83]^ | - | - | - | 4 ^[15, 44]^ |
|  | Phenytoine | 4 | - | - | - | 2 ^[32, 131]^ | 2^[15]^ |
|  | Topiramate | 4 | 2 ^[74]^ | - | 1 ^[4]^ | 1 ^[99]^ | - |
|  | Phenobarbital | 2 | - | - | - | - | 2 ^[15]^ |
|  | Vigabatrin | 2 | - | - | 2 ^[97]^ | - | - |
|  | Clobazam | 1 | - | - | - | - | 1 ^[44]^ |
| Anti-parkinsonians | L-dopa | 28 | - | - | - | - | 28 ^[15, 72, 136]^ |
|  | Dopamine agonists | 8 | - | - | - | - | 8 ^[15, 136]^ |
|  | Non-competitive (NMDA)-glutamate receptor-antagonist (amantadine) (also see ‘anti-dementia’) | 10 | 2 ^[50]^ | - |  | 1 ^[143]^ | 7 ^[15, 89]^ |
|  | COMT inhibitors | 1 | - | - | - | - | 1 ^[15]^ |
|  | MAO-inhibitors | 1 | - | - | - | - | 1 ^[15]^ |
| Anesthetics | General anesthetics | 42 | 1 ^[135]^ | 15 ^[144]^ | 8 ^[71, 144]^ | 7 ^[144]^  ^[76]^ | 11^[15, 49, 82]^ |
|  | Local anesthetics | 4 | - | - | 4 ^[3, 21, 78]^ | - | - |
| Anti-dementia | Cholinesterase inhibitors | 18 | - | - | - | - | 18 ^[15]^ |
|  | Non-competitive (NMDA)-glutamate receptor-antagonist (memantine) (also see anti-parkinsonians) | 9 | - | 1^[107]^ | - | 3 ^[12, 95]^ | 5 ^[70, 93, 103]^ |
| Cytostatics | Ifosfamide | 5 | - | - | 1 ^[91]^ | 4 ^[118]^ | - |
|  | Prednimustine | 4 | - | - | 3 ^[87],[94]^ | 1 ^[87]^ | - |
|  | Chlorambucil | 2 | - | - | 1 ^[142]^ | - | 1 ^[142]^ |
| Others | Anti-emetics | 23 | 1 ^[60]^ | 1 ^[96]^ | 2 ^[23]^ | - | 19 ^[15]^ |
|  | Anti-arrhythmics | 5 | - | - | 3 ^[24, 28, 137]^ | 1 ^[129]^ | 1^[15]^ |
|  | Vitamins | 5 | - | - | 4 ^[48, 101, 146]^ | 1 ^[102]^ | - |
|  | Anti-hypertensives | 2 | - | - | 2 ^[134, 140]^ | - | - |
|  | Contrast agents | 3 | 1 ^[41]^ | - | 2 ^[10, 19, 81]^ | - | - |
|  | Immunomodulating drugs | 2 | - | - | 1 ^[39]^ | - | 1 ^[15]^ |
|  | Anti-fibrinolytic agents | 1 | - | - | 1 ^[58]^ | - | - |
|  | Anti-histamines | 1 | - | - | - | 1 ^[61]^ | - |
|  | Anti-hypotensives | 1 | - | - | - | - | 1 ^[141]^ |
|  | Anti-tussives | 1 | - | - | - | 1 ^[127]^ | - |
|  | Adrenergic bronchodilators | 3 | - | - | 3 ^[92]^ | - | - |
|  | NSAID | 1 | - | - | - | - | 1 ^[15]^ |
|  | Anti-viral agents |  |  |  | 1 ^[45]^ |  |  |
|  | Anti-malaria prophylaxis |  |  |  | 1 ^[64]^ |  |  |

Classes and subclasses of drugs described to cause drug-induced myoclonus. References were sorted to distribution of myoclonus. The numbers of reported cases of drug-induced myoclonus are listed. ‘-’ no studies describing myoclonus with this distribution, for this class of drugs.

**References**

1. Adair JC, el-Nachef A, Cutler P (1996) Fentanyl neurotoxicity. Ann Emerg Med 27:791-792

2. Aguglia U, Gambardella A, Zappia M, Valentino P, Quattrone A (1995) Negative myoclonus during valproate-related stupor. Neurophysiological evidence of a cortical non-epileptic origin. Electroencephalogr Clin Neurophysiol 94:103-108

3. Alfa JA, Bamgbade OA (2008) Acute myoclonus following spinal anaesthesia. Eur J Anaesthesiol 25:256-257

4. Alonso-Navarro H, Jimenez-Jimenez FJ (2006) Reversible tremor, myoclonus, and fasciculations associated with topiramate use for migraine. Clin Neuropharmacol 29:157-159

5. Asconape J, Diedrich A, DellaBadia J (2000) Myoclonus associated with the use of gabapentin. Epilepsia 41:479-481

6. Askenasy JJ, Yahr MD (1988) Is monoamine oxidase inhibitor induced myoclonus serotoninergically mediated? J Neural Transm 72:67-76

7. Babiy M, Stubblefield MD, Herklotz M, Hand M (2005) Asterixis related to gabapentin as a cause of falls. Am J Phys Med Rehabil 84:136-140

8. Bak TH, Bauer M, Schaub RT, Hellweg R, Reischies FM (1995) Myoclonus in patients treated with clozapine: a case series. J Clin Psychiatry 56:418-422

9. Barak Y, Levine J, Weisz R (1996) Clozapine-induced myoclonus: two case reports. J Clin Psychopharmacol 16:339-340

10. Barbaccia JJ (1995) Myoclonus in the postanesthesia care unit after an intraoperative myelogram. Anesth Analg 80:413-414

11. Batra YK, Rajeev S, Lokesh VC, Rao KL (2007) Spinal myoclonus associated with intrathecal bupivacaine and fentanyl in an infant. Can J Anaesth 54:587-588

12. Bougea A, Gerakoulis S, Anagnostou E, Paraskevas G, Kapaki E, Kararizou E (2014) Donepezil-induced myoclonus in a patient with Alzheimer disease. Ann Pharmacother 48:1659-1661

13. Bowdle TA (1987) Myoclonus following sufentanil without EEG seizure activity. Anesthesiology 67:593-595

14. Bowdle TA, Rooke GA (1994) Postoperative myoclonus and rigidity after anesthesia with opioids. Anesth Analg 78:783-786

15. Brefel-Courbon C, Gardette V, Ory F, Montastruc JL (2006) Drug-induced myoclonus: a French pharmacovigilance database study. Neurophysiol Clin 36:333-336

16. Bruera E, Pereira J (1997) Acute neuropsychiatric findings in a patient receiving fentanyl for cancer pain. Pain 69:199-201

17. Cartwright PD, Hesse C, Jackson AO (1993) Myoclonic spasms following intrathecal diamorphine. J Pain Symptom Manage 8:492-495

18. Casas M, Garcia-Ribera C, Alvarez E, Udina C, Queralto JM, Grau JM (1987) Myoclonic movements as a side-effect of treatment with therapeutic doses of clomipramine. Int Clin Psychopharmacol 2:333-336

19. Casazza M, Bracchi M, Girotti F (1985) Spinal myoclonus and clinical worsening after intravenous contrast medium in a patient with spinal arteriovenous malformation. AJNR Am J Neuroradiol 6:965-966

20. Caviness JN, Evidente VG (2003) Cortical myoclonus during lithium exposure. Arch Neurol 60:401-404

21. Celik Y, Bekir Demirel C, Karaca S, Kose Y (2003) Transient segmental spinal myoclonus due to spinal anaesthesia with bupivacaine. J Postgrad Med 49:286

22. Chan S, Turner MR, Young L, Gregory R (2006) Cephalosporin-induced myoclonus. Neurology 66:E20

23. Chaw SH, Chan L, Lee PK, Bakar JA, Rasiah R, Foo LL (2016) Prolonged drug-induced myoclonus: is it related to palonosetron? J Anesth

24. Chua TP, Farrell T, Lipkin DP (1994) Myoclonus associated with propafenone. BMJ 308:113

25. Crespel A, Genton P, Berramdane M, Coubes P, Monicard C, Baldy-Moulinier M, Gelisse P (2005) Lamotrigine associated with exacerbation or de novo myoclonus in idiopathic generalized epilepsies. Neurology 65:762-764

26. de Armendi AJ, Fahey M, Ryan JF (1993) Morphine-induced myoclonic movements in a pediatric pain patient. Anesth Analg 77:191-192

27. De Conno F, Caraceni A, Martini C, Spoldi E, Salvetti M, Ventafridda V (1991) Hyperalgesia and myoclonus with intrathecal infusion of high-dose morphine. Pain 47:337-339

28. Deik AF, Shanker VL (2012) A case of amiodarone-associated myoclonus responsive to levetiracetam. Can J Neurol Sci 39:680-681

29. Devanand DP, Sackeim HA, Brown RP (1988) Myoclonus during combined tricyclic antidepressant and lithium treatment. J Clin Psychopharmacol 8:446-447

30. Dib EG, Bernstein S, Benesch C (2004) Multifocal myoclonus induced by trimethoprim-sulfamethoxazole therapy in a patient with nocardia infection. N Engl J Med 350:88-89

31. Dominguez C, Benito-Leon J, Bermejo-Pareja F (2009) Multifocal myoclonus induced by haloperidol. Neurol Sci 30:385-386

32. Duarte J, Sempere AP, Cabezas MC, Marcos J, Claveria LE (1996) Postural myoclonus induced by phenytoin. Clin Neuropharmacol 19:536-538

33. Dutra LA, Pedroso JL, Felix EP, Barsottini OG (2008) Venlafaxine induced-myoclonus in a patient with mixed dementia. Arq Neuropsiquiatr 66:894-895

34. Ege F, Kocak Y, Titiz AP, Ozturk SM, Ozturk S, Ozbakir S (2008) Gabapentin-Induced myoclonus: case report. Mov Disord 23:1947-1948

35. Eisele JH, Jr., Grigsby EJ, Dea G (1992) Clonazepam treatment of myoclonic contractions associated with high-dose opioids: case report. Pain 49:231-232

36. Essandoh S, Sakae M, Miller J, Glare PA (2010) A cautionary tale from critical care: resolution of myoclonus after fentanyl rotation to hydromorphone. J Pain Symptom Manage 40:e4-6

37. Evidente VG, Caviness JN (1999) Focal cortical transient preceding myoclonus during lithium and tricyclic antidepressant therapy. Neurology 52:211-213

38. Farrington J, Stoudemire A, Tierney J (1995) The role of ciprofloxacin in a patient with delirium due to multiple etiologies. Gen Hosp Psychiatry 17:47-53

39. Ferbert A, Biniek R, Kindler J, Maurin N (1993) Myoclonus and tremor induced acutely by administration of tumor necrosis factor in a patient with Ehlers-Danlos syndrome. Mov Disord 8:232-233

40. Fernandez Corcuera P, Pomarol E, Amann B, McKenna P (2008) Myoclonus provoked by lamotrigine in a bipolar patient. J Clin Psychopharmacol 28:248-249

41. Finsterer J, Lubec D, Verlicchi A, Samec P (1998) Facial myocloni and stroke as late sequelae of metrizamide myelography. J Neuropsychiatry Clin Neurosci 10:472-473

42. Fukuzako H, Tominaga H, Izumi K, Koja T, Nomoto M, Hokazono Y, Kamei K, Fujii H, Fukuda T, Matsumoto K (1990) Postural myoclonus associated with long-term administration of neuroleptics in schizophrenic patients. Biol Psychiatry 27:1116-1126

43. Garvey MJ, Tollefson GD (1987) Occurrence of myoclonus in patients treated with cyclic antidepressants. Arch Gen Psychiatry 44:269-272

44. Genton P, Nguyen VH, Mesdjian E (1998) Carbamazepine intoxication with negative myoclonus after the addition of clobazam. Epilepsia 39:1115-1118

45. Gentry JL, 3rd, Peterson C (2015) Death Delusions and Myoclonus: Acyclovir Toxicity. Am J Med 128:692-694

46. George M, Haasz M, Coronado A, Salhanick S, Korbel L, Kitzmiller JP (2013) Acute dyskinesia, myoclonus, and akathisa in an adolescent male abusing quetiapine via nasal insufflation: a case study. BMC Pediatr 13:187

47. Ghaziuddin N, Iqbal A, Khetarpal S (2001) Myoclonus during prolonged treatment with sertraline in an adolescent patient. J Child Adolesc Psychopharmacol 11:199-202

48. Grech V, Vella C, Mercieca V (2001) Temporary myoclonus with treatment of congenital transcobalamin 2 deficiency. Pediatr Neurol 24:75-76

49. Greenberg M, Hilty C (2003) Myoclonus after prolonged infusion of etomidate treated with dantrolene. J Clin Anesth 15:489-490

50. Gupta A, Lang AE (2010) Drug-induced cranial myoclonus. Mov Disord 25:2264-2265

51. Hagen N, Swanson R (1997) Strychnine-like multifocal myoclonus and seizures in extremely high-dose opioid administration: treatment strategies. J Pain Symptom Manage 14:51-58

52. Han PK, Arnold R, Bond G, Janson D, Abu-Elmagd K (2002) Myoclonus secondary to withdrawal from transdermal fentanyl: case report and literature review. J Pain Symptom Manage 23:66-72

53. Healy DG, Ingle GT, Brown P (2009) Pregabalin- and gabapentin-associated myoclonus in a patient with chronic renal failure. Mov Disord 24:2028-2029

54. Herishanu YO, Zlotnik M, Mostoslavsky M, Podgaietski M, Frisher S, Wirguin I (1998) Cefuroxime-induced encephalopathy. Neurology 50:1873-1875

55. Hofmann A, Tangri N, Lafontaine AL, Postuma RB (2006) Myoclonus as an acute complication of low-dose hydromorphone in multiple system atrophy. J Neurol Neurosurg Psychiatry 77:994-995

56. Holtkamp M, Halle A, Meierkord H, Masuhr F (2006) Gabapentin-induced severe myoclonus in a patient with impaired renal function. J Neurol 253:382-383

57. Horga G, Horga A, Baeza I, Castro-Fornieles J, Lazaro L, Pons A (2010) Drug-induced speech dysfluency and myoclonus preceding generalized tonic-clonic seizures in an adolescent male with schizophrenia. J Child Adolesc Psychopharmacol 20:233-234

58. Hui AC, Wong TY, Chow KM, Szeto CC (2003) Multifocal myoclonus secondary to tranexamic acid. J Neurol Neurosurg Psychiatry 74:547

59. Huppertz HJ, Feuerstein TJ, Schulze-Bonhage A (2001) Myoclonus in epilepsy patients with anticonvulsive add-on therapy with pregabalin. Epilepsia 42:790-792

60. Immovilli P, Rota E, Morelli N, Iafelice I, Magnacavallo A, Guidetti D (2015) Metoclopramide-induced facial and palatopharyngeal myoclonus. Neurology 84:1284

61. Irioka T, Machida A, Yokota T, Mizusawa H (2008) Antihistamine-associated myoclonus: A case report. Mov Disord 23:1615-1616

62. Ito S, Liao S (2008) Myoclonus associated with high-dose parenteral methadone. J Palliat Med 11:838-841

63. Jayathissa S, Woolley M, Ganasegaram M, Holden J, Cu E (2010) Myoclonus and delirium associated with ciprofloxacin. Age Ageing 39:762

64. Jimenez-Huete A, Gil-Nagel A, Franch O (2002) Multifocal myoclonus associated with mefloquine chemoprophylaxis. Clin Neuropharmacol 25:243

65. Jundt F, Lempert T, Dorken B, Pezzutto A (2004) Trimethoprim-sulfamethoxazole exacerbates posthypoxic action myoclonus in a patient with suspicion of Pneumocystis jiroveci infection. Infection 32:176-178

66. Kango Gopal G, Hewton C, Pazhvoor SK (2014) Myoclonus associated with concomitant ciprofloxacin and oxycodone in an older patient. British journal of clinical pharmacology 77:906-907

67. Kaufman KR, Parikh A, Chan L, Bridgeman M, Shah M (2014) Myoclonus in renal failure: Two cases of gabapentin toxicity. Epilepsy & behavior case reports 2:8-10

68. Kettl P, DePaulo JR, Jr. (1983) Maprotiline-induced myoclonus. J Clin Psychopharmacol 3:264-265

69. Khasani S (2015) Cefepime-induced jaw myoclonus. Neurology 84:1183

70. Kitagawa N, Takeuchi A (2014) Memantine-induced myoclonus. Neurology 83:1387

71. Kiyama S, Yoshikawa T (1998) Persistent intraoperative myoclonus during propofol-fentanyl anaesthesia. Can J Anaesth 45:283-284

72. Klawans HL, D'Amico DJ, Patel BC (1975) Behavioral supersensitivity to 5-hydroxytryptophan induced by chronic methysergide pretreatment. Psychopharmacologia 44:297-300

73. Kloke M, Bingel U, Seeber S (1994) Complications of spinal opioid therapy: myoclonus, spastic muscle tone and spinal jerking. Support Care Cancer 2:249-252

74. Kutluay E, Pakoz B, Beydoun A (2007) Reversible facial myoclonus with topiramate therapy for epilepsy. Epilepsia 48:2001-2002

75. Lau KK, Kink RJ, Jones DP (2004) Myoclonus associated with intraperitoneal imipenem. Pediatr Nephrol 19:700-701

76. Laughlin TP, Newberg LA (1985) Prolonged myoclonus after etomidate anesthesia. Anesth Analg 64:80-82

77. Lauterbach EC (1999) Hiccup and apparent myoclonus after hydrocodone: review of the opiate-related hiccup and myoclonus literature. Clin Neuropharmacol 22:87-92

78. Lin CS, Wei-Hung C, Lee YW (2008) Transient spinal myoclonus after spinal anaesthesia with bupivacaine in the perioperation period. Anaesthesist 57:518

79. Lippmann S, Moskovitz R, O'Tuama L (1977) Tricyclic-induced myoclonus. Am J Psychiatry 134:90-91

80. Lippmann S, Tucker D, Wagemaker H, Schulte T (1977) A second report of tricyclic-induced mycolonus. Am J Psychiatry 134:585-586

81. Lu CS, Chu NS (1988) Acute dystonic reaction with asterixis and myoclonus following metoclopramide therapy. J Neurol Neurosurg Psychiatry 51:1002-1003

82. Lumley J, Morgan M (1985) Myoclonus after etomidate anesthesia. Anesth Analg 64:1034

83. Magaudda A, Di Rosa G (2012) Carbamazepine-induced non-epileptic myoclonus and tic-like movements. Epileptic Disord 14:172-173

84. Magny JF, d'Allest AM, Nedelcoux H, Zupan V, Dehan M (1994) Midazolam and myoclonus in neonate. Eur J Pediatr 153:389-390

85. Man BL, Fu YP (2015) Piperacillin/tazobactam-induced myoclonic jerks in a man with chronic renal failure. BMJ Case Rep 2015

86. Marinella MA (2001) Myoclonus and generalized seizures associated with gatifloxacin treatment. Arch Intern Med 161:2261-2262

87. Martin M, Diaz-Rubio E, Casado A, Valverde JJ, Garcia Urra D, Lopez-Martin JA, Rodriguez-Lescure A (1994) Prednimustine-induced myoclonus--a report of three cases. Acta Oncol 33:81-82

88. Masand P (1992) Desipramine-induced oral-pharyngeal disturbances: stuttering and jaw myoclonus. J Clin Psychopharmacol 12:444-445

89. Matsunaga K, Uozumi T, Qingrui L, Hashimoto T, Tsuji S (2001) Amantadine-induced cortical myoclonus. Neurology 56:279-280

90. McKeon A, Pittock SJ, Glass GA, Josephs KA, Bower JH, Lennon VA, Ahlskog JE (2007) Whole-body tremulousness: isolated generalized polymyoclonus. Arch Neurol 64:1318-1322

91. Meyer T, Ludolph AC, Munch C (2002) Ifosfamide encephalopathy presenting with asterixis. J Neurol Sci 199:85-88

92. Micheli F, Cersosimo MG, Scorticati MC, Velez M, Gonzalez S (2000) Myoclonus secondary to albuterol (salbutamol) instillation. Neurology 54:2022-2023

93. Moellentin D, Picone C, Leadbetter E (2008) Memantine-induced myoclonus and delirium exacerbated by trimethoprim. Ann Pharmacother 42:443-447

94. Monnerat C, Gander M, Leyvraz S (1997) A rare case of prednimustine-induced myoclonus. J Natl Cancer Inst 89:173-174

95. Murgai AA, LeDoux MS (2015) Memantine-induced Myoclonus in a Patient with Alzheimer Disease. Tremor and other hyperkinetic movements 5:337

96. Nampiaparampil D, Oruc NE (2006) Metodopramide-induced palatopharyngeal myoclonus. Mov Disord 21:2028-2029

97. Neufeld MY, Vishnevska S (1995) Vigabatrin and multifocal myoclonus in adults with partial seizures. Clin Neuropharmacol 18:280-283

98. Olszewska DA, Chalissery AJ, Williams J, Lynch T, Smyth S (2015) Speech myoclonus due to probable pregabalin adverse drug-reaction. Parkinsonism & related disorders 21:823-824

99. Oulis P, Potagas C, Masdrakis VG, Thomopoulos Y, Kouzoupis AV, Soldatos CR (2008) Reversible tremor and myoclonus associated with topiramate-fluvoxamine coadministration. Clin Neuropharmacol 31:366-367

100. Ozcan B, Kavurt S, Yucel H, Bas AY, Demirel N (2015) Rhythmic myoclonic jerking induced by midazolam in a preterm infant. Pediatr Neurol 52:e9

101. Ozdemir O, Baytan B, Gunes AM, Okan M (2010) Involuntary movements during vitamin B12 treatment. J Child Neurol 25:227-230

102. Ozer EA, Turker M, Bakiler AR, Yaprak I, Ozturk C (2001) Involuntary movements in infantile cobalamin deficiency appearing after treatment. Pediatr Neurol 25:81-83

103. Papageorgiou SG, Kontaxis T, Antelli A, Kalfakis N (2007) Exacerbation of myoclonus by memantine in a patient with Alzheimer disease. J Clin Psychopharmacol 27:407-408

104. Patel HC, Bruza D, Yeragani V (1988) Myoclonus with trazodone. J Clin Psychopharmacol 8:152

105. Patel S, Roshan VR, Lee KC, Cheung RJ (2006) A myoclonic reaction with low-dose hydromorphone. Ann Pharmacother 40:2068-2070

106. Patterson JF (1990) Myoclonus caused by a tricyclic antidepressant. South Med J 83:463-465

107. Pei LJ, Tianzhi IL, Lim WS (2015) Memantine-Induced Myoclonus Precipitated by Renal Impairment and Drug Interactions. Journal of the American Geriatrics Society 63:2643-2644

108. Potter JM, Reid DB, Shaw RJ, Hackett P, Hickman PE (1989) Myoclonus associated with treatment with high doses of morphine: the role of supplemental drugs. BMJ 299:150-153

109. Praharaj SK, Venkatesh BG, Sarkhel S, Zia-ul-Haq M, Sinha VK (2010) Clozapine-induced myoclonus: a case study and brief review. Prog Neuropsychopharmacol Biol Psychiatry 34:242-243

110. Reutens DC, Stewart-Wynne EG (1989) Norpethidine induced myoclonus in a patient with renal failure. J Neurol Neurosurg Psychiatry 52:1450-1451

111. Rivera M, Crespo M, Teruel JL, Marcen R, Ortuno J (1999) Neurotoxicity due to imipenem/cilastatin in patients on continuous ambulatory peritoneal dialysis. Nephrol Dial Transplant 14:258-259

112. Rosen JB, Milstein MJ, Haut SR (2012) Olanzapine-associated myoclonus. Epilepsy Res 98:247-250

113. Rosenhagen MC, Schmidt U, Weber F, Steiger A (2006) Combination therapy of lamotrigine and escitalopram may cause myoclonus. J Clin Psychopharmacol 26:346-347

114. Rozan JP, Kahn CH, Warfield CA (1995) Epidural and intravenous opioid-induced neuroexcitation. Anesthesiology 83:860-863

115. Sackellares JC, Smith DB (1979) Myoclonus with electrocerebral silence in a patient receiving penicillin. Arch Neurol 36:857-858

116. Sarhill N, Davis MP, Walsh D, Nouneh C (2001) Methadone-induced myoclonus in advanced cancer. Am J Hosp Palliat Care 18:51-53

117. Sarva H, Panichpisal K (2012) Gentamicin-induced myoclonus: a case report and literature review of antibiotics-induced myoclonus. Neurologist 18:385-388

118. Savica R, Rabinstein AA, Josephs KA (2011) Ifosfamide associated myoclonus-encephalopathy syndrome. J Neurol 258:1729-1731

119. Scullin P, Sheahan P, Sheila K (2003) Myoclonic jerks associated with gabapentin. Palliat Med 17:717-718

120. Shea YF, Mok MM, Chang RS (2014) Gabapentin-induced myoclonus in an elderly with end-stage renal failure. Journal of the Formosan Medical Association = Taiwan yi zhi 113:660-661

121. Sjogren P, Jonsson T, Jensen NH, Drenck NE, Jensen TS (1993) Hyperalgesia and myoclonus in terminal cancer patients treated with continuous intravenous morphine. Pain 55:93-97

122. Sjogren P, Thunedborg LP, Christrup L, Hansen SH, Franks J (1998) Is development of hyperalgesia, allodynia and myoclonus related to morphine metabolism during long-term administration? Six case histories. Acta Anaesthesiol Scand 42:1070-1075

123. Sonck J, Laureys G, Verbeelen D (2008) The neurotoxicity and safety of treatment with cefepime in patients with renal failure. Nephrol Dial Transplant 23:966-970

124. Spina Silva T, Dal-Pra Ducci R, Zorzetto FP, Braatz VL, de Paola L, Kowacs PA (2014) Meropenem-induced myoclonus: a case report. Seizure 23:912-914

125. Striano P, Zara F, Coppola A, Ciampa C, Pezzella M, Striano S (2007) Epileptic myoclonus as ciprofloxacin-associated adverse effect. Mov Disord 22:1675-1676

126. Takahashi T, Masuya Y, Ueno K, Watanabe K, Takahashi M, Morita S, Higashima M, Wada Y (2015) Clozapine-related negative myoclonus associated with urinary tract infection: a case report. J Clin Psychopharmacol 35:205-206

127. Tanaka A, Nagamatsu T, Yamaguchi M, Nomura A, Nagura F, Maeda K, Tomino T, Watanabe T, Shimizu H, Fujita Y, Ito Y (2011) Myoclonus after dextromethorphan administration in peritoneal dialysis. Ann Pharmacother 45:e1

128. Thwaites D, McCann S, Broderick P (2004) Hydromorphone neuroexcitation. J Palliat Med 7:545-550

129. Ting SM, Lee D, Maclean D, Sheerin NS (2008) Paranoid psychosis and myoclonus: flecainide toxicity in renal failure. Cardiology 111:83-86

130. Tominaga H, Fukuzako H, Izumi K, Koja T, Fukuda T, Fujii H, Matsumoto K, Sonoda H, Imamura K (1987) Tardive myoclonus. Lancet 1:322

131. Trauner DA (1985) Stimulus-induced myoclonus and burst suppression on EEG: effects of phenytoin toxicity. Ann Neurol 17:312-313

132. Tremolizzo L, Fermi S, Fusco ML, Susani E, Frigo M, Piolti R, Ferrarese C, Appollonio I (2011) Generalized action myoclonus associated with escitalopram in a patient with mixed dementia. J Clin Psychopharmacol 31:394-395

133. Uchihara T, Tsukagoshi H (1988) Myoclonic activity associated with cefmetazole, with a review of neurotoxicity of cephalosporins. Clin Neurol Neurosurg 90:369-371

134. Vadlamudi L, Wijdicks EF (2002) Multifocal myoclonus due to verapamil overdose. Neurology 58:984

135. Van Keulen SG, Burton JH (2003) Myoclonus associated with etomidate for ED procedural sedation and analgesia. Am J Emerg Med 21:556-558

136. Vardi J, Glaubman H, Rabey JM, Streifler M (1978) Myoclonic attacks induced by L-dopa and bromocryptin in Parkinson patients: a sleep EEG study. J Neurol 218:35-42

137. Velasco SL, Sierra-Hidalgo F, Rodriguez RM, Guerreo AJ, Morales JR (2014) Flecainide-induced myoclonus. Clin Neuropharmacol 37:65-66

138. Velayudhan L, Kirchner V (2005) Quetiapine-induced myoclonus. Int Clin Psychopharmacol 20:119-120

139. Vural A, Tezer FI (2012) Myoclonus induced by haloperidol in the intensive care unit. J Neuropsychiatry Clin Neurosci 24:E41

140. Wallace EL, Lingle K, Pierce D, Satko S (2009) Amlodipine-induced myoclonus. Am J Med 122:e7

141. Wierre L, Decaudin B, Barsumau J, Vairon MX, Horrent S, Odou P, Azar R (2004) Dobutamine-induced myoclonia in severe renal failure. Nephrol Dial Transplant 19:1336-1337

142. Wyllie AR, Bayliff CD, Kovacs MJ (1997) Myoclonus due to chlorambucil in two adults with lymphoma. Ann Pharmacother 31:171-174

143. Yarnall AJ, Burn DJ (2012) Amantadine-induced myoclonus in a patient with progressive supranuclear palsy. Age Ageing 41:695-696

144. Yates AM, Wolfson AB, Shum L, Kehrl T (2013) A descriptive study of myoclonus associated with etomidate procedural sedation in the ED. Am J Emerg Med 31:852-854

145. Yoon JH, Lee PH, Yong SW, Park HY, Lim TS, Choi JY (2008) Movement disorders at a university hospital emergency room. An analysis of clinical pattern and etiology. J Neurol 255:745-749

146. Zanus C, Alberini E, Costa P, Colonna F, Zennaro F, Carrozzi M (2012) Involuntary movements after correction of vitamin B12 deficiency: a video-case report. Epileptic Disord 14:174-180

147. Zhang C, Glenn DG, Bell WL, O'Donovan CA (2005) Gabapentin-induced myoclonus in end-stage renal disease. Epilepsia 46:156-158
