# Supplementary material for: The clinical heterogeneity of drug-induced myoclonus: an illustrated review
Source: J Neurol. 2016 Dec 16;264(8):1559–66. doi: 10.1007/s00415-016-8357-z (PMC5533847; doi:10.1007/s00415-016-8357-z)
Supplement: Supplementary file 2 — Supplementary material 2 (DOCX 11 kb) [file 415_2016_8357_MOESM2_ESM.docx]

**Video legends**

Video 1: Case A, propriospinal myoclonus induced by antibiotics

79-year-old woman with myoclonus of the trunk, abdomen and arms (particularly right shoulder) more than legs due to ciprofloxacin and/or penicillin.

Video 2: Case B, generalized myoclonus induced by amantadine

66-year-old man with generalized myoclonus due to the NMDA antagonist amantadine. Myoclonus predominates in his face and neck, affecting his speech. Axial myoclonus is apparent during walking.
